# Morphology and Compressive Properties of Extruded Polyethylene Terephthalate Foam

**DOI:** 10.3390/polym16060776

**Published:** 2024-03-12

**Authors:** Zhicheng Zhang, Chunling Xin, Chiyuan Ma, Wenchong Xu, Feng Ren, Yadong He

**Affiliations:** College of Mechanical and Electronically Engineering, Beijing University of Chemical Technology, Beijing 100029, China; 2019400138@buct.edu.cn (Z.Z.); xincl@mail.buct.edu.cn (C.X.); 2022210356@buct.edu.cn (C.M.); 2021200655@buct.edu.cn (W.X.); renfeng@mail.buct.edu.cn (F.R.)

**Keywords:** extruded PET foam, anisotropy, aspect ratio, compressive properties, elongated Kelvin model, rectangular model

## Abstract

The cell structure and compressive properties of extruded polyethylene terephthalate (PET) foam with different densities were studied. The die of the PET foaming extruder is a special multi-hole breaker plate, which results in a honeycomb-shaped foam block. The SEM analysis showed that the aspect ratio and cell wall thickness of the strand border is greater than that of the strand body. The cells are elongated and stronger in the extruding direction, and the foam anisotropy of the structure and compressive properties decrease with increasing density. The compression results show typical stress–strain curves even though the extruded PET foam is composed of multiple foamed strands. The compression properties of PET foam vary in each of the three directions, with the best performing direction (i.e., extrusion direction) showing stretch-dominated structures, while the other two directions show bending-dominated structures. Foam mechanics models based on both rectangular and elongated Kelvin cell geometries were considered to predict the compressive properties of PET foams in terms of relative density, structure anisotropy, and the properties of the raw polymer. The results show that the modulus and strength anisotropy of PET foam can be reasonably predicted by the rectangular cell model, but more accurate predictions were obtained with an appropriately assumed elongated Kelvin model.

## 1. Introduction

Polymer foams are commonly used in various industries, including packaging, construction, wind turbine blades, automotive, and aerospace industries, due to their numerous advantages. These include their controllability, abundance of raw materials, affordability, high specific strength and modulus, thermal insulation, impact resistance, and noise reduction capabilities [1,2,3]. Polyethylene terephthalate (PET) foam has several advantages, including a non-toxic manufacturing process, easy recyclability, high heat deflection temperature, relatively good mechanical performance, and lower cost raw materials, or the use of recycled PET material. PET foam is increasingly replacing thermoplastic rigid foams like polycarbonate (PC) and polyvinyl chloride (PVC) in structural applications, such as wind turbine blades, construction, and automotive applications, due to its advantages [1,4,5].

The foaming process plays an important role in the foam structure, and foam performance is directly influenced by its structure [6,7,8,9,10,11]. PET’s low melt strength and viscoelasticity make it difficult to be foamed, which has been extensively discussed in the literature [12,13,14,15]. Extrusion foaming is a popular method for polymer foaming due to its continuous production and high productivity, and low-cost PET foam is commonly extruded for commercial applications [13,16,17,18]. In order to increase the PET foaming ratio and foam thickness, the multi-hole die has been developed for the extrusion foaming process [19,20,21]. Due to the limited spacing between the holes in the die, the individual expanded PET foaming strands are quickly compressed into a uniform foam block when they pass through the holes [19]. These processes give PET foam a unique morphology (open-cell or close-cell) and meso-structure (honeycomb-shaped foam) [1,19,20]. 

The compressive stress–strain behavior of foams has been systematically studied by Gibson et al. [6,7]. Subsequent researchers have also conducted numerous studies on various specific foams [22,23,24,25,26]. The compressive behavior of foams is typically divided into three states: the linear elastic region, plateau region, and densification region, as shown in second section [6,27,28]. According to Ashby’s latest work, the deformation of elastoplastic foams, whether open-cell or closed-cell, can be divided into two main categories: stretch-dominated structures and bending-dominated structures [29]. The mechanical properties of bending-dominated structures are weaker than those of stretch-dominated structures under the same density level. For the bending-dominated case, the so-called soft modes in the deformation mechanisms are usually involved, while the hard modes such as axial compression and stretching contribute to the stretch-dominated structures [29]. 

Extrusion polymer foams have a tendency to be elongated in the extrusion direction, which is characterized by the shape anisotropy (aspect ratio) *R*. The aspect ratio was defined as the average ratio of the cell size in the parallel and perpendicular directions of elongation [9,30]. The foam’s structure anisotropy results in mechanical anisotropy. To establish the relationship between the structural anisotropy and performance anisotropy of foams, a rectangular model has been proposed by Gibson et al. [6,9]. They simplified the foam cell structure to a rectangular model, investigated the relationship between the structural anisotropy and mechanical anisotropy of foams, and developed a corresponding analytical theoretical model [6]. However, Gibson’s model was conducted by an ideal rectangular cell structure, which over-simplifies the cell structure of real foam. Some of the results in the literature suggest that over-simplified models can result in significant discrepancies between predicted and experimental values [2,24,30,31,32,33]. To obtain a more accurate model, Sullivan et al. developed an elongated Kelvin (tetrakaidecahedron) model to describe the relationship between the structural anisotropy and mechanical anisotropy [34,35,36,37]. To define the cell geometry in the elongated Kelvin model, in addition to the shape anisotropy *R*, a shape parameter *Q* is required. The elongated Kelvin model is much closer to the real cell geometry than the rectangular model and more accurate [2,30,31,32]. Although the elongated Kelvin model was developed for the open-cell foam, foam with a thinner face may also be acceptable [31,32].

Mazzuca et al. conducted a study on the mechanical behavior of PET foam under shear and compression at elevated temperatures using a comprehensive experimental and analytical methodology, and the results show that the shear and compression properties of PET foam experience a significant reduction as the temperature increases, particularly when it approaches or exceeds the Tg [38]. Yao et al. conducted a study on the effect of crystallization on the tensile properties of PET foam using both experimental and modelling approaches. The results indicate that increasing the crystalline phase content leads to an improvement in the material’s tensile properties [1]. Taliabue et al. developed an image-based approach to investigate the structure of polymer foams based on PET foam and create 3D numerical models. Their work revealed general connections between foam microstructure and mechanical characteristics [28]. Fathi et al. conducted a systematic study on the morphology and mechanical properties of extruded PET foams at various length scales. The study investigated the mechanical properties and multi-scale morphology of strand PET foams. The relationship between deformation phenomena at different scales and the resulting macroscopic mechanical properties was fundamentally established by Fathi et al. [20].

The relationship between the morphology and mechanical properties of polymeric foams such as PVC, polyurethane (PU), and polypropylene (PP) has been extensively studied [3,8,9,10,23,24,39,40]. The mechanical properties of PET foam in relation to its morphology have been little studied. In this work, the microstructure and compression properties of PET foam with four different densities in three directions were determined experimentally. Whether the unique honeycomb-shaped internal structure has an effect on the global compressive stress–strain of PET foam is discussed in this work, as well as the deformation mechanisms. The relationship between the anisotropy of the PET foam cell structure and the anisotropy of the PET foam compressive properties is discussed and the accuracy of the elongated Kelvin model and the rectangular model for predicting structural anisotropy and compressive anisotropy is also evaluated.

## 2. Materials and Methods

### 2.1. Materials

**Figure 1:**
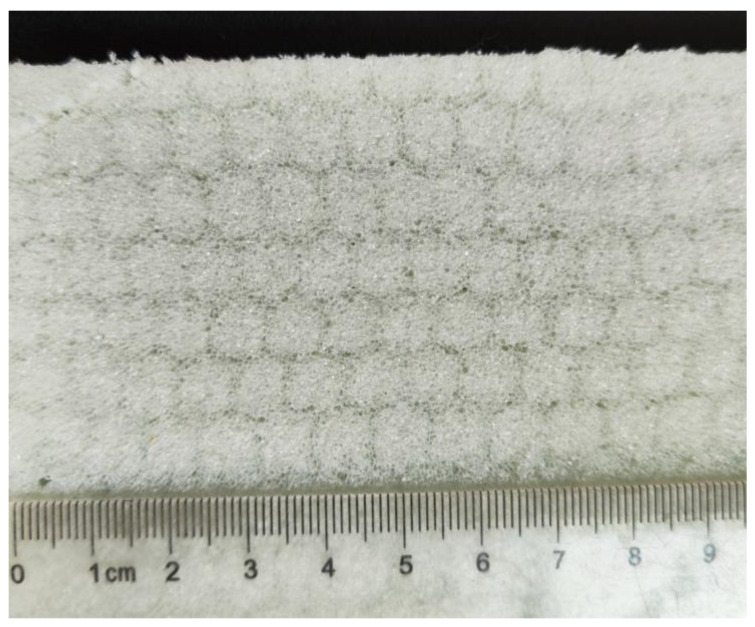
Extruded PET foam composed of multi-foamed strands.

**Figure 2:**
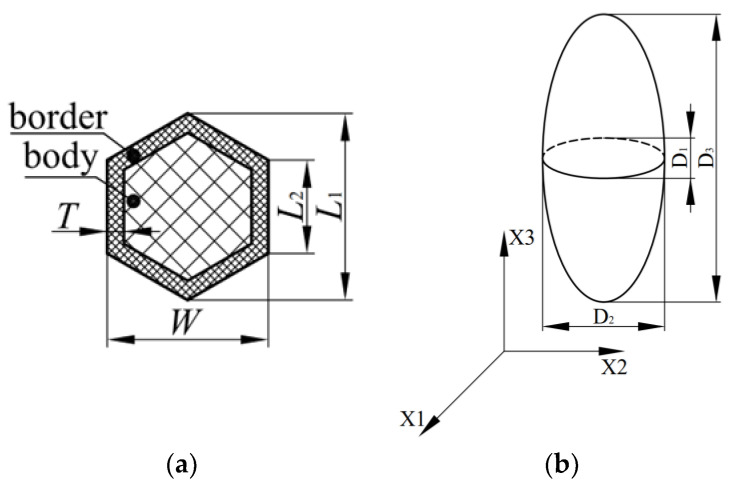
(**a**) PET foam strand unit; (**b**) coordinates of PET foam.

The closed-cell polyethylene terephthalate (PET) foam used in this work was manufactured by PolyCore Advanced Materials Co., Ltd in Huangshan, China. The honeycomb-shaped foam shown in Figure 1 was produced using the strand foam extrusion process. The die of the PET foaming extruder is a special multi-hole breaker plate [19,20], as discussed in the introduction. PET foam typically exhibits higher performance in the processing direction due to stretching during extrusion, compared to other directions. The strand consists of two parts: the high-density strand border and the low-density strand body (see Figure 2a) [20]. *L*_1_, *L*_2_, *T*, and *W* are the geometric features of the stand border. In Figure 2b, the extrusion direction is defined as X3, and the lowest performance is defined as X1. D1, D2, and D3 are the geometric features of the cell in the extrusion and lateral directions, respectively. The foaming process has been discussed in detail [19,20], and four different densities of PET foam, namely YD100, YD150, YD200, and YD320, are investigated in this work. The relevant information is shown in Table 1. 

### 2.2. Morphological Characterization

Most polymeric foams exhibit structural anisotropy due to processing methods, and the morphology of PET foams has been carefully examined using a scanning electron microscope (SEM) with magnifications of X20, X30, X40, and X50. The SEM is TM4000, which was purchased from Hitachi, Ltd in Tokyo, Japan. The SEM images were analyzed using ImageJ 1.54i. The cell diameter, aspect ratio, and sphericity were measured in the X3X1 and X2X1 planes of the PET foam. The cell wall thickness in the X2X1 plane of the PET foam was also considered in the work. According to Zhou Yong’ findings, for more accurate statistical results, the statistics on cell diameter, aspect ratio, and sphericity are all based on 300 cells [41]. The cell shape in the low-density PET foam is more likely to be a tetradecahedron [20]; the cell size is evaluated by measuring the equivalent diameter due to the irregular shape of the cell. The equivalent diameter was calculated using Formula (1) [42]:

(1) $$D_{e}=\frac{1}{n}\sum_{i=1}^{n}2\sqrt{S∕\pi }$$

where the *D*_e_ is the equivalent diameter and *S* is the area of the cell. 

The method used to determine the cell aspect ratio refers to [32]:

(2) $$R_{j1}=\frac{1}{n}\sum_{i=1}^{n}\frac{D_{j}}{D_{1}}$$

where the *R_j_*_1_ is the aspect ratio and *j =* 2, 3 corresponds to the X2 and X3 directions in Figure 2. 

The sphericity reflects the shape of cell in a foam, and the sphericity was calculated following [42]:

(3) $$S_{p}=\frac{1}{n}\sum_{i=1}^{n}\frac{4\pi S}{P^{2}}$$

where the *S*_P_ is the sphericity and *P* is the perimeter of the cell. 

The relative density was calculated as follows:

(4) $$\rho_{r}=\frac{\rho_{f}}{\rho_{s}}$$

where the *ρ*_f_ is the density of the foam and *ρ*_s_ is the density of the solid PET polymer. 

Cell edge thickness was measured in this work, according to the method in [23]. The thickness of the planes of X2X1 of the PET foam with four different densities were measured. Approximately 100 measurements were taken at the border and body of the PET foam strand. 

### 2.3. Compression Tests

Compression tests were performed according to ISO844-2021 [43] using a SANS CMT5105 universal tester. The PET foam was cut into 50 mm × 50 mm × 50 mm blocks. The test speed was 5 mm/min, and all samples were tested in a controlled environment at a temperature of 23 °C and 50% relative humidity. In order to obtain more accurate results, five replicates were measured. 

**Figure 3:**
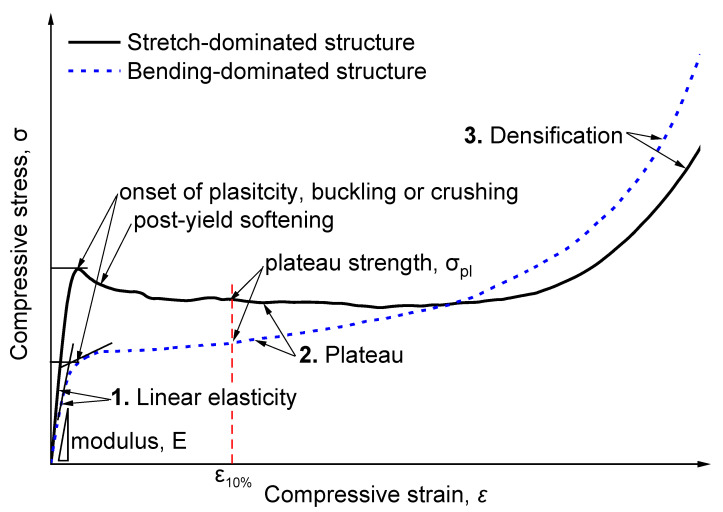
Typical compressive stress–strain curve for stretch-dominated structures and bending-dominated structures.

Figure 3 shows the typical compressive stress–strain curve of foams, which has been discussed extensively. The curves can be divided into three different regions, i.e., the elastic region, plateau region, and densification region [2,8,24,40]. The compressive modulus was determined by calculating the slope of the initial linear elasticity region [25], as shown in Figure 3. The plateau strength of the PET foam was determined as the stress at 10% strain [20,44]. To identify whether cell structures are stretch-dominated or bending-dominated, many phenomenological and micromechanical models indicate that the relative modulus and plateau stress’s dependence on relative density follows a power law [6,29]. The most well-known relations are those by Ashby [29]:

(5) $$\frac{E_{f}}{E_{s}}\propto {\left(\frac{\rho_{f}}{\rho_{s}}\right)}^{n}$$

(6) $$\frac{\sigma_{pl}}{\sigma_{s}}\propto {\left(\frac{\rho_{f}}{\rho_{s}}\right)}^{n}$$

*n* will be close to 1 if the cell wall’s axial compression or stretching are the main deformation mechanisms (e.g., buckling of cell walls); if the cell wall’s bending is the main deformation mechanism, *n* approaches 2 [29,45]. Stretch-dominated structures usually undergo initial yielding, followed by local brittle collapse or plastic buckling of the cell structure. This revealed the post-yield softening phenomena in their stress–strain response (see Figure 3) [20,29]. 

### 2.4. Anisotropic Models

**Figure 4:**
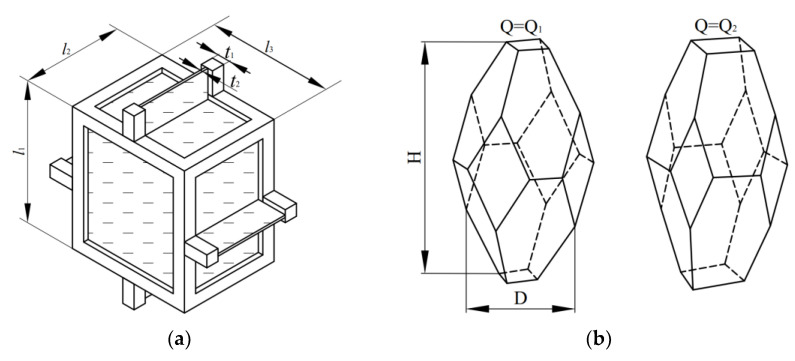
Repeated cell unit: (**a**) rectangular model; (**b**) elongated Kelvin model.

As discussed in the introduction, the rectangular cell model (Figure 4a) has been widely used to predict the anisotropy of foam properties. Gibson has conducted a lot of work on the rectangular cell models; for the closed-cell foam, the elastic modulus and plateau strength anisotropy models can be related using Equations (7) and (8) [6].

(7) EjE1=ϕ2Rj121+1Rj123+1−ϕ2Rj121+1Rj12

(8) σjσ1=2Rj11+1Rj1

where *φ* is the cell edge content of foam cell and *R_j_*_1_ is the aspect ratio (shape anisotropy), *j* = 2, 3. The measurement of the cell edge and face is crucial for accurately determining the rectangular model for elastic modulus anisotropy. However, measuring *φ* can be challenging, and some scholars have found that experimental results differ significantly from the model predictions [25,46,47]. According to Gibson’s research, the plateau strength anisotropy is not affected by the cell edge content [6]. 

The model proposed by Gibson and Ashby oversimplifies the cell structure of real foam, leading to significant discrepancies between the model’s predictions and experimental results [2,30,31,32,33]. To improve the precision of the model predictions, Sullivan et al. have refined the Kelvin foam model. They have developed an elongated tetrakaidecahedron model (elongated Kelvin model) that is more similar to the foam structure [34,35,36,37]. The predictions of the model were consistent with the experimental results [2,30,31,32,33]. In contrast to the cell structure anisotropy *R*, the elongated Kelvin model requires a second term, the shape parameter *Q*, to define the cell geometry. Figure 4b illustrates how *Q* affects the cell geometry. In Figure 4b, both cells have the same aspect ratio, but Q_1_ is smaller than Q_2_. The elastic modulus and plateau strength anisotropy models can be related using Equations (9) and (10). Both the rectangular and elongated Kelvin models assume that the foam’s basic constituent material is isotropic [6,34]:

(9) $$R_{j1}^{E}=\frac{{\left(R_{j1}\right)}^{2}}{4}\left[\frac{\left(2P^{2}{\left(R_{j1}\right)}^{2}+\frac{64Q^{3}}{\sqrt{16+P^{2}{\left(R_{j1}\right)}^{2}}}\right)C_{1}+\frac{8R_{j1}C_{2}P^{3}\left(32+4Q\sqrt{16+P^{2}{\left(R_{j1}\right)}^{2}}\right)\rho_{r}}{\left(4Q+2\sqrt{16+P^{2}{\left(R_{j1}\right)}^{2}}\right)\left(16+P^{2}{\left(R_{j1}\right)}^{2}\right)}}{16C_{1}+\frac{8{\left(R_{j1}\right)}^{3}C_{2}P^{5}}{\left(4Q+2\sqrt{16+P^{2}{\left(R_{j1}\right)}^{2}}\right)\left(16+P^{2}{\left(R_{j1}\right)}^{2}\right)}\rho_{r}}\right]$$

(10) $$R_{j1}^{\sigma }=R_{j1}\left[\frac{\sqrt{C_{1}}PR_{j1}+\frac{16\sqrt{2}C_{3}P^{1.5}{\left(R_{j1}\right)}^{0.5}{\rho_{r}}^{0.5}}{{\left(4Q+2\sqrt{16+P^{2}{\left(R_{j1}\right)}^{2}}\right)}^{0.5}{\left(16+P^{2}{\left(R_{j1}\right)}^{2}\right)}^{0.5}}}{4\sqrt{C_{1}}+\frac{4\sqrt{2}C_{3}P^{2.5}{\left(R_{j1}\right)}^{1.5}{\rho_{r}}^{0.5}}{{\left(4Q+2\sqrt{16+P^{2}{\left(R_{j1}\right)}^{2}}\right)}^{0.5}{\left(16+P^{2}{\left(R_{j1}\right)}^{2}\right)}^{0.5}}}\right]$$

where *j* = 2, 3, $P=2+\sqrt{2}Q$, $C_{1}=\sqrt{3}-\pi /2$, $C_{2}=\left(20\sqrt{3}-11\pi \right)/\left(2\sqrt{3}-\pi \right)$, $C_{3}=\left(60-11\sqrt{3}\pi \right)/24\left(\sqrt{3}-\pi /2\right)$ for a hypocycloid cross-section.

## 3. Results and Discussion

### 3.1. Cell Structure Anisotropy

**Figure 5:**
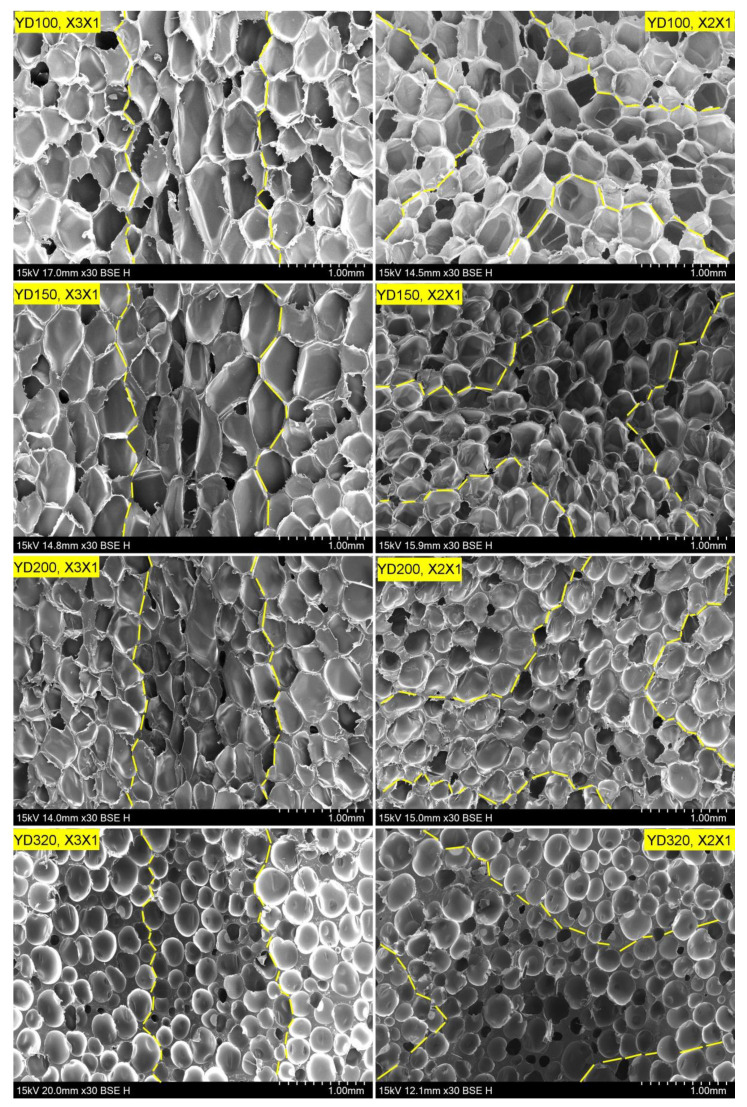
SEM micrographs of strand border and strand body in X3X1 plane and X2X1 plane for PET foam with four different densities. The cells between the yellow lines represent the strand border portion, while the remaining part is the strand body.

Although the foam structure is stochastic in nature, there are several regular characteristics. The structure of the cell has a significant effect on the foam properties. The equivalent diameter of the cells, their aspect ratio, and sphericity of the honeycomb-shaped PET foam are discussed in this section. Figure 5 shows representative SEM micrographs of the foam with four different densities, providing an intuitive view of the cell structure. The cells between the yellow lines represent the strand border portion, while the remaining part is the strand body. Compared to the X2X1 plane, the cell is more elongated in the X3X1 plane, and the cell shows significant structural anisotropy. This is expected as the cells are stretched in the extrusion direction during the foaming process. In addition, the cells in the strand border are slenderer than those in the strand body at the same plane. Strands expand due to the sudden release of pressure as they are extruded through the multi-hole die. The transverse space is limited, so the bubble grows faster in the extrusion direction. Simultaneously, the temperature on the surface of the strand is lower, resulting in the rapid cooling and shaping of the bubbles. However, the bubbles inside the strand are not fully cooled and shaped. These two factors may be responsible for the cells in the strand border appearing more elongated than the cells in the strand body. It is difficult to compare the size of the cells in different regions of the low-density foam from the SEM, such as YD100, YD150, and YD200. However, the YD320 cells in the strand body are significantly larger than those in the strand border. The same situation is observed with respect to cell thickness, and Table 2 shows the quantitative results. Although the cell edge thickness varies greatly across all densities, it tends to increase as density increases [23]. Low-density foams usually have a tetrahedral cell structure, as observed in YD100 and YD150 [20]. As density increases, the cells tend to become more rounded. In the case of the YD320 foam stand body, the cells are more elliptical and have fewer angles. Bubble growth mechanisms show that bubbles initially grow spherically and squeeze at high foaming ratios [48,49]. Thomson’s research has shown that the tetrakaidecahedron is the only polyhedron that can pack to fill space and minimize surface area per unit volume [6,34,50,51]. This may explain why the cells at a low foaming ratio are round with thicker walls, while the cells at a high foaming ratio tend to be more tetrahedral with thinner walls. 

**Figure 6:**
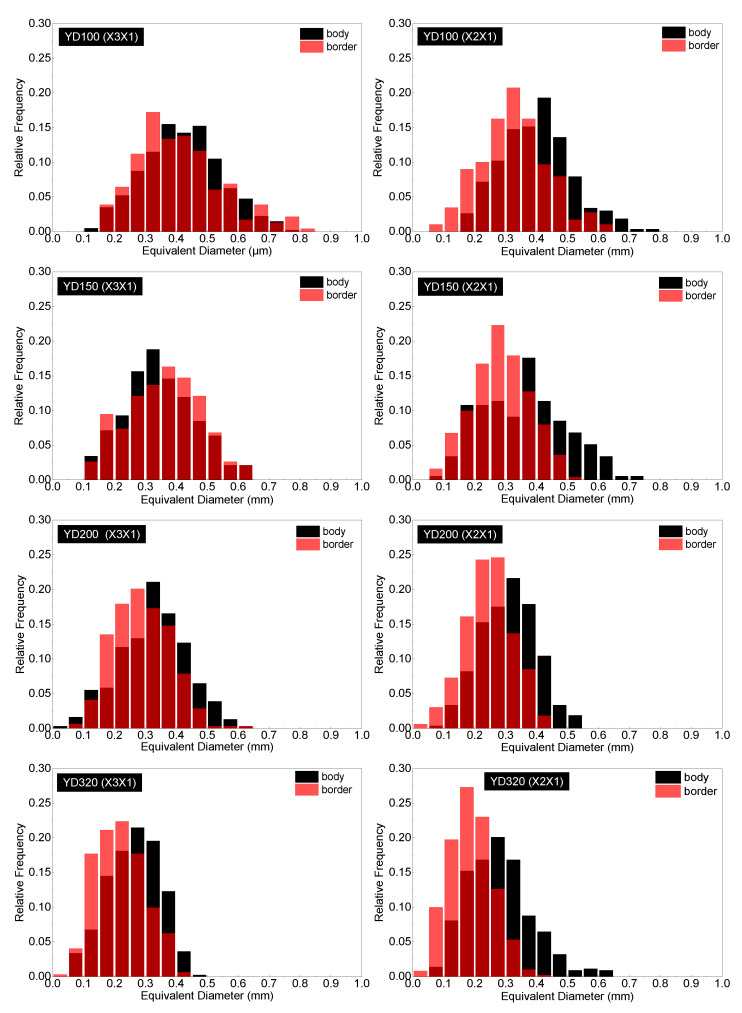
Cell equivalent diameter distributions in the strand border and strand body for YD100, YD150, YD200, and YD320 foam samples in the X3X1 and X2X1 planes. Red columns are cells in the strand border; black columns are cells in the strand body. The dark red are the overlap between the cell equivalent diameter distributions of the strand body and border.

**Figure 7:**
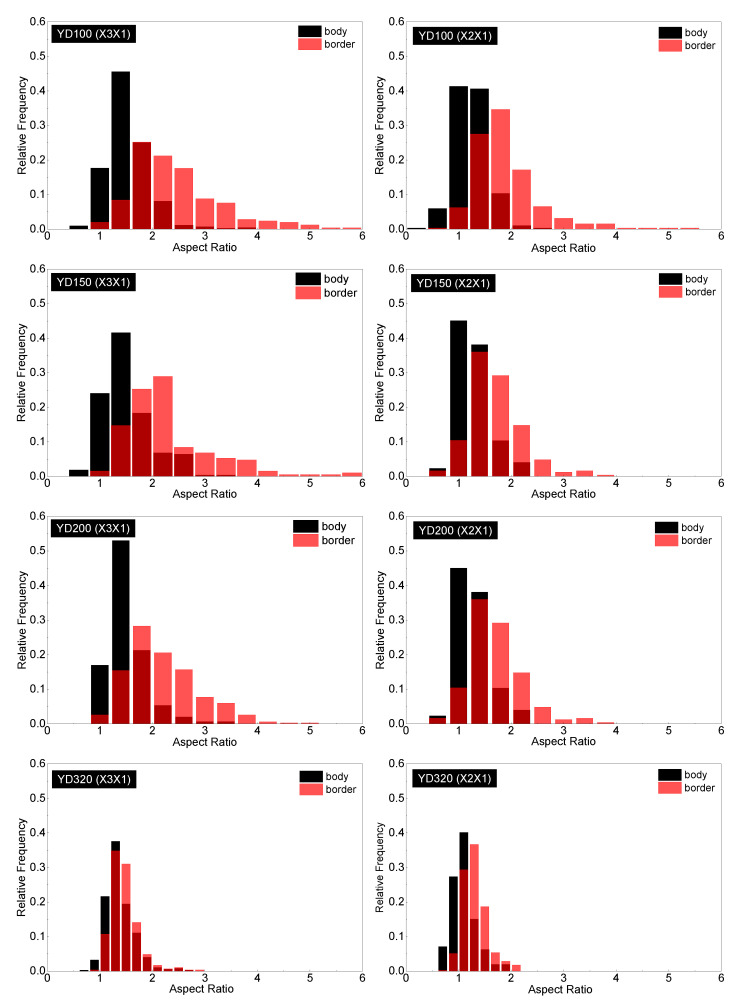
Aspect ratio distributions in the strand border and strand body for YD100, YD150, YD200, and YD320 foam samples in the X3X1 and X2X1 planes. Red columns are cells in the strand border; black columns are cells in the strand body. The dark red are the overlap between the aspect ratio distributions of the strand body and border.

**Figure 8:**
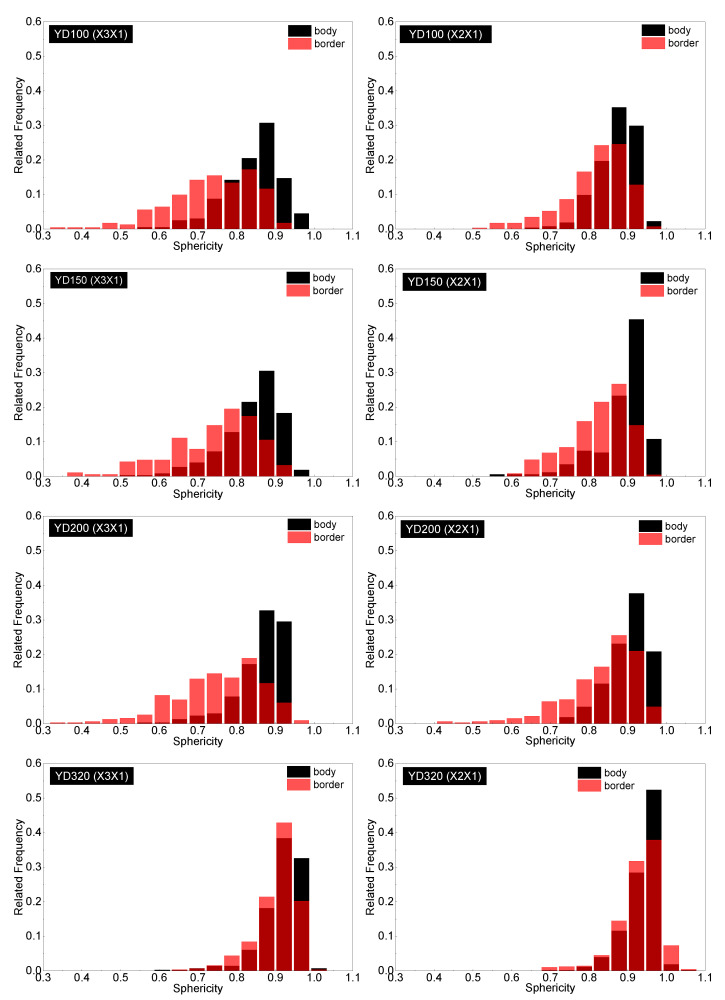
Sphericity distributions in the strand border and strand body for YD100, YD150, YD200, and YD320 foam samples in the X3X1 and X2X1 planes. Red columns are cells in the strand border; black columns are cells in the strand body. The dark red are the overlap between the sphericity distributions of the strand body and border.

**Figure 9:**
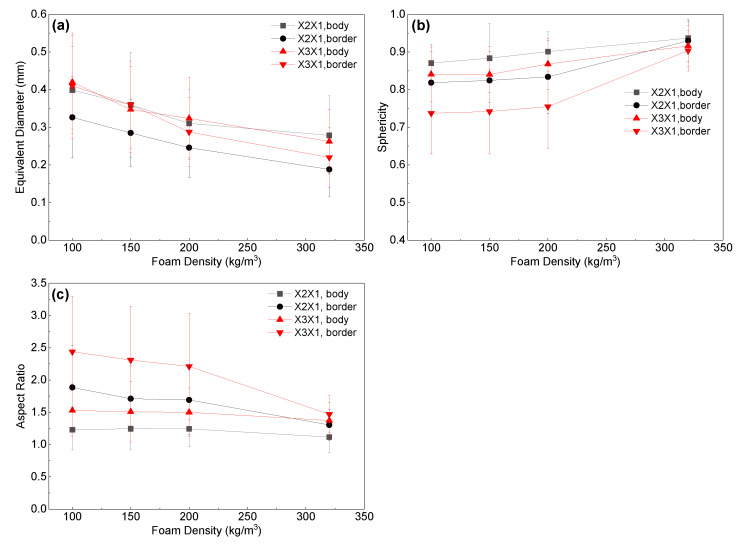
Foam structural features in the strand border and body: (**a**) Equivalent diameter vs. foam density, (**b**) sphericity vs. foam density, and (**c**) aspect ratio vs. foam density.

**Figure 10:**
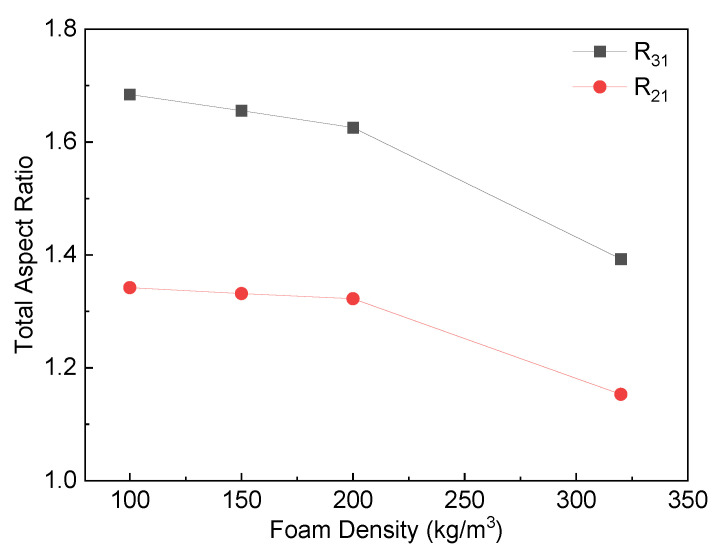
Total aspect ratio of YD100, YD150, YD200, and YD320 PET foam samples as a function of foam density in the X3X1 plane (*R*_31_) and the X2X1 plane (*R*_21_).

Due to the complex geometrical features of the PET foam, SEM graphs present the apparent results. To conduct a quantitative analysis of the cell structure, Figure 6, Figure 7 and Figure 8 display the distributions of cell equivalent diameter, aspect ratio, and sphericity for the strand border and strand body in planes X3X1 and X2X1. Figure 6 and Figure 9a confirm that the cell diameter decreases as the foam density increases. YD100 foam has an equivalent diameter of approximately 400 microns, while YD320 foam has an equivalent diameter of only about 200 microns. The low-density PET foams (YD100 and YD150) have a broad cell size distribution, whereas YD320 has a narrow cell size distribution. Overall, the size of the cells follows a Gaussian distribution. In the X3X1 plane, the cell size and distribution width of the cells in the strand body and strand border of the YD100 and YD150 foam samples are comparable, as shown in Figure 6 and Figure 9a. In addition, the size of the cells in the strand body are larger than those in the strand border, and the width of the cell size distribution at the body is comparable to or slightly larger than that at the border. Smaller cells and thicker cell walls mean that density is higher at the strand border than in the strand body, which has been demonstrated in [20]. Figure 7 and Figure 9c show the distributions of the cell aspect ratio for both the strand body and strand border of the PET foam with four different densities; all the aspect ratios obey Gamma distribution, and similar patterns have been reported by other researchers [41]. The aspect ratio and distribution width of the cells in the strand body are smaller than those in strand border. The foam block composed of extruded foam strands is typically used to carry the load, rather than using the strands individually. This paper analyses the compression performance of the foam from a macroscopic perspective. The total aspect ratio of the PET foam block should be calculated based on the area fraction of the cells [20]. The total aspect ratio of X3X1 plane or X2X1 plane was calculated as follows:

(11) $$R_{j1}=a_{f}R_{j1}^{1}+\left(1-a_{f}\right)R_{j1}^{2}$$

where the *R_j_*_1_ is the total aspect ratio, *j* = 2, 3, *a*_f_ is the strand border area fraction of the strand, *R*^1^*_j_*_1_ is the aspect ratio of the strand border, and *R*^2^*_j_*_1_ is the aspect ratio of the strand body. The strand border area fraction is shown in Table 3, and the total aspect ratio is shown in Figure 10. The total aspect ratio in the X3X1 plane is 1.68, 1.66, 1.63, and 1.39 with increasing density, while the total aspect ratio in the X2X1 plane is 1.34, 1.33, 1.32, and 1.15 with increasing density. As the density of the foam increases, the total aspect ratio of both the X3X1 plane and the X2X1 plane decreases, and the total aspect ratio of X3X1 is greater than X2X1 at the same density. As shown in Figure 8 and Figure 9b, all the sphericities follow Weibull distribution in the strand body and strand border of the PET foam with four different densities [41]. Whether in the X3X1 plane or X2X1 plane, the cell sphericity in the strand body is greater than that in the strand border, while the distribution width is narrow. The sphericity increases with increasing density, meaning that the cells in the high-density foam are closer to spheres, which is consistent with the SEM analysis discussed above.

### 3.2. Compression Properties

**Figure 11:**
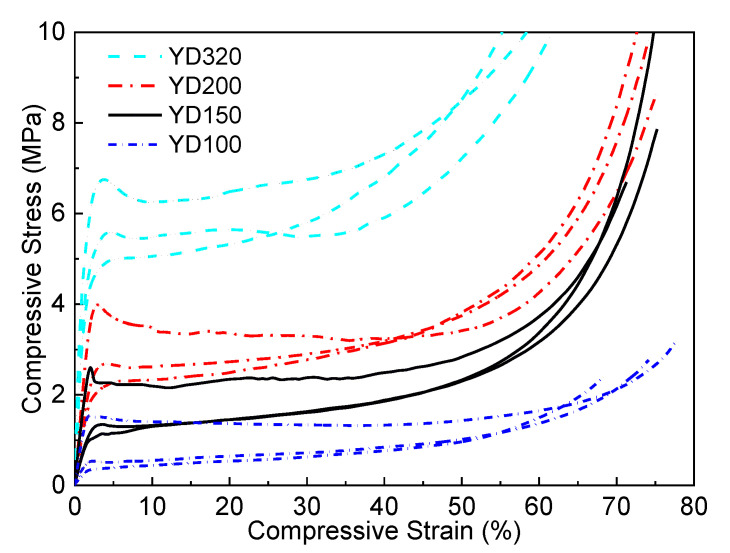
Compression stress–strain curves of YD100, YD150, YD200, and YD320 PET foam samples in X1, X2, and X3 directions. Highest performance is X3 direction; lowest performance is X1 direction.

**Figure 12:**
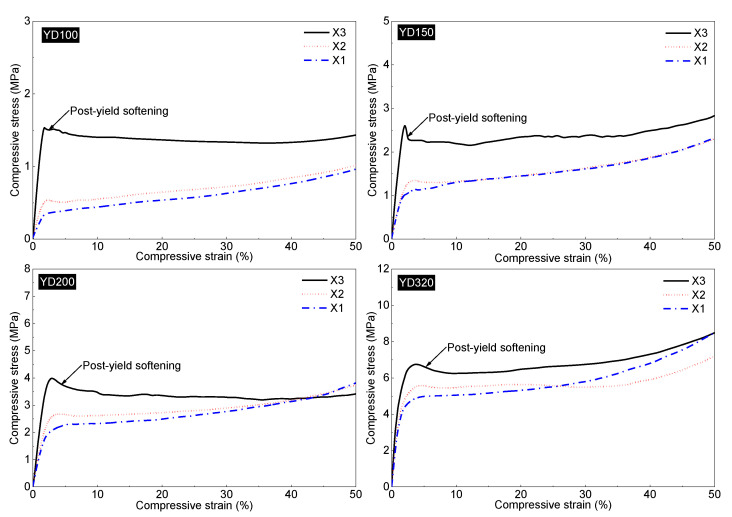
Partial compression stress–strain curves of YD100, YD150, YD200, and YD320 PET foam samples in X1, X2, and X3 directions. Black line is X3 direction; red line is X2 direction; and blue line is X1 direction.

**Figure 13:**
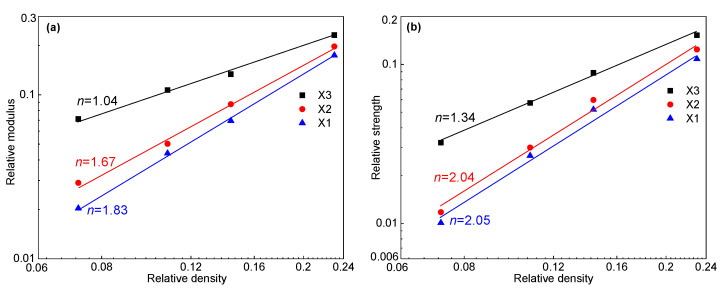
The power law fits the experimental relative modulus and experimental relative strength as a function of relative density in three directions using Equations (5) and (6): (**a**) relative modulus vs. relative density; (**b**) relative strength vs. relative density.

**Figure 14:**
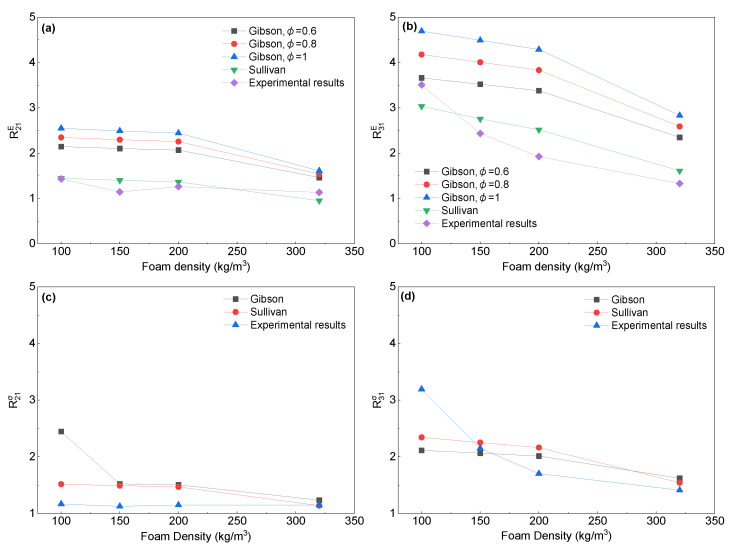
Experimental strength and modulus anisotropy vs. model predictions obtained by Gibson’ model, Equations (7) and (8), and Sullivan’s model, Equations (9) and (10): (**a**) modulus anisotropy of X2X1plane; (**b**) modulus anisotropy of X3X1plane; (**c**) strength anisotropy of X2X1 plane; and (**d**) strength anisotropy of X3X1 plane.

Figure 11 shows the global compression stress–strain curves of four PET foams in the X1, X2, and X3 directions. Compressive stress–strain curves for the four PET foams in three directions are generally similar to those reported in the literature [2,8,24,40,52]. The curves can be divided into three different regions, i.e., the elastic region, plateau region, and densification region. Although PET foam exists with a honeycomb-shaped strand border, the global stress–strain curves demonstrate typical characteristics, which has been proved in conventional metal and polymer foams [6,7,29]. It also shows that extruded PET foams, although composed of foamed strands, can still be analyzed from a macroscopic point of view in terms of the relationship between the mechanical properties of the foam and its structure. Obviously, the foam mechanical properties improve with higher density. This has been extensively covered in the references [6,25,53]. But there has been little discussion of whether the curves are bending-dominated or stretch-dominated. Figure 11 shows that the strength and modulus of the X3 direction are significantly higher than those of the other two directions. The X1 direction exhibits the worst performance. The foam’s unique morphology may be responsible for its much higher compressive strength and modulus in the X3 direction. Firstly, the foam’s honeycomb-shaped structure improves the mechanical properties in this direction [20]. At the same time, as analyzed above, the cell is elongated and has a higher aspect ratio than those in X2 direction, resulting in a high-performance strength-dominated structure. The compressive properties in the X2 direction are little higher than those in the X1 direction. This may be due to the insignificant elongation of the foam in the X2 direction, as illustrated in Figure 10. The global *R*_21_ of the four PET foams is greater than 1. As shown in Figure 12, a distinct post-yield softening appears in the strain–stress curves of the foam in the X3 direction, indicating that the cell structures in the X3 direction are stretch-dominated structures, while the cell structures in the X1 direction are bending-dominated [29]. Stress in the X2 direction is slightly higher than that in the X1 direction at a low strain, and also exhibits insignificant post-yield softening. According to Ashby’s findings [29], the value of *n* in Equations (5) and (6) is 1 for stretch-dominated structures, and the value of *n* is 2 for bending-dominated structures. Since PET foam raw materials are not known due to commercial confidentiality requirements [20], solid PET with a density of 1.39 g/cm^3^, yield strength of 45 Mpa, and Young’s modulus of 1.6 Gpa was assumed. The power law lines fitted to the data in Figure 13 provide several useful pieces of information. The results demonstrate that the modulus and strength in the X1 direction and X2 direction can be fitted by Equations (5) and (6) with *n* values close to 2. This finding aligns with the previously discussed theories, which suggest a typical bending-dominated structure in the X1 and X2 directions. Meanwhile, the fitted line for the X3 direction yields *n* values within the range of 1, which suggests a stronger stretch-dominated structure. As the density increases, the compressive modulus and strength become more comparable in all directions, converging towards the same values. This indicates that the anisotropy of compressive properties decreases with increasing density. Figure 10 and Figure 14 demonstrate that both the anistropic compressive properties and structural anisotropy decrease as the foam density increases. This suggests a strong correlation between the structural anisotropy and anistropic compressive properties.

### 3.3. Anisotropy Analysis

To investigate the relationship between the anisotropy of the cell structure and the anisotropy of the compressive properties, we predicted the anisotropy ratios of the compressive properties using both Gibson’s theory (rectangular cell model) and Sullivan’s theory (elongated Kelvin cell model or tetrakaidecahedron cell model) [6,34]. For the rectangular cell model, only the measurements of the aspect ratio are necessary to obtain the anisotropy of the compression properties. When considering the elongated Kelvin model, it is important to take into account not only the aspect ratio of the cell but also its geometry parameter *Q*. In the previous work, *Q* was restricted to 1, 2, and $\sqrt{2}$, and the value of *Q* that better described the morphology of the cells is 1 in the present work. The prediction of strength anisotropy for the rectangular cell model is not affected by the cell edge content *φ*. However, the prediction of modulus anisotropy requires an accurate measurement of *φ*. The difficulty of measuring the cell edge content has been discussed in several publications, as outlined in the introduction [54]. Furthermore, distinguishing the boundaries between the edge and in the SEM can be challenging [24]. For closed-cell foam, the cell edge is usually considered to make up the major portion in rectangular model [6]. Figure 14 indicates that the modulus anisotropy of the foam was predicted using the assumed cell edge content series. A comparison of both the experimental results and model predictions of the modulus anisotropy and strength anisotropy is shown in Figure 14. The rectangular model tended to overestimate the experimental results for both strength and modulus anisotropy. However, the predicted trends were generally consistent with the experimental results. The rectangular model provides a reasonably accurate prediction of strength anisotropy. However, for all given cell edge contents, the model shows a higher degree of deviation in predicting the modulus anisotropy. The rectangular model assumption that the cross-section of the cell structure is square and the size of the edges and faces is constant may not be appropriate for most polymer foams [55]. Therefore, estimating the modulus and strength anisotropy using the rectangular model may result in imprecise outcomes [24]. Meanwhile, the experimental results for both the modulus and strength anisotropy are consistent with the theoretical predictions of the elongated Kelvin model with minimal error, which is also supported by other studies [30,31,32]. This can be attributed to the fact that the elongated Kelvin model approximates the real foam microstructure, providing the analytical model with a more accurate description of the actual cellular morphology of PET foams. 

## 4. Conclusions

Extruded PET foams with four different densities were investigated in the three directions. The cell microstructure was captured using a SEM and the characteristics of the cell structure were studied in detail. Quasi-static tests were used to determine the compressive properties in the three directions, and the deformation behavior was discussed. Cellular mechanics models based on both rectangular and elongated Kelvin cell geometries were considered to predict the compression properties of PET foams. The following conclusions can be drawn:

1. Although the foam structure is stochastic in nature, there are some regular characteristics. The cell structure is greatly influenced by the extrusion foaming process. Extrusion direction cells have a larger aspect ratio, equivalent diameter, and lower sphericity. The aspect ratio and equivalent circular diameter decrease as the foam density increases, while the cell wall thickness and sphericity show the opposite trend. Compared to the cells in the strand body, the cells in strand border have a larger aspect ratio, thicker cell wall thickness, and lower sphericity, making the extruded PET foam resemble a honeycomb structure.
2. The PET foam compressive stress–strain curves demonstrate typical characteristics, which has been proved in conventional metal and polymer foams. Although the extruded PET foams are composed of foamed strands, they can still be analyzed from a macroscopic point of view in terms of the relationship between the mechanical properties of the foam and its structure. The cells in the extrusion direction (X3 direction) undergo the most significant stretching phenomenon and demonstrate the best compressive properties. The cells in the X2 direction exhibit some stretching, and their compressive properties are slightly better than those in the X1 direction. Ashby’s theory suggests that the structures in the X1 and X2 directions are bending-dominated, while the structures in the X3 direction are stretch-dominated. A strong correlation was discovered between the anisotropy of the cell shape and compressive properties.
3. The elongated Kelvin model provides a more accurate description of the actual cell morphology of PET foams, and experimental results for both modulus and strength anisotropy are consistent with the theoretical predictions of this model. Gibson’s model was conducted by an ideal rectangular cell structure, which oversimplifies the cell structure of real foam; the strength and modulus anisotropy may be reasonably predicted by the rectangular cell model. The elongated Kelvin mode can be used to analyze extruded PET foam.

## Figures and Tables

**Table 1:** Nominal densities of PET foam samples.

PET Foam	Density (kg/m^3^)
YD100	100.12 ± 1.72
YD150	154.58 ± 2.84
YD200	204.51 ± 3.19
YD320	321.37 ± 3.55

**Table 2:** Cell thickness of PET foam.

PET Foam	Strand Border Thickness (μm)	Strand Body Thickness (μm)
YD100	7.46 ± 3.29	7.24 ± 3.40
YD150	13.13 ± 6.78	10.90 ± 5.63
YD200	22.43 ± 13.53	16.26 ± 8.28
YD320	34.59 ± 18.14	29.77 ± 17.76

**Table 3:** Strand structural features.

Foam	*L*_1_ (mm)	*L*_2_ (mm)	*W* (mm)	*T* (μm)	*a*_f_ (%)
YD100	9.83 ± 0.26	6.73 ± 0.19	7.11 ± 0.25	736.4 ± 85	17.07
YD150	9.75 ± 0.34	6.47 ± 0.23	7.08 ± 0.37	789.4 ± 93	18.35
YD200	10.23 ± 0.29	6.35 ± 0.30	6.91 ± 0.21	762.4 ± 69	17.66
YD320	9.67 ± 0.27	6.71 ± 0.18	6.87 ± 0.17	830.8 ± 107	19.65

## Data Availability

Data are contained within the article.
